# Supplementary material for: Mapping Stress-Responsive Signaling Pathways Induced by Mitochondrial Proteostasis Perturbations
Source: bioRxiv. 2024 Feb 1:2024.01.30.577830. Preprint. [Version 1] doi: 10.1101/2024.01.30.577830 (PMC10862789; doi:10.1101/2024.01.30.577830)
Supplement: Supplement 2 [file NIHPP2024.01.30.577830v1-supplement-2.pdf]

bioRxiv preprint doi: https://doi.org/10.1101/2024.01.30.577830; this version posted February 1, 2024. The copyright holder for this preprint (which was not certified by peer review) is the author/funder, who has granted bioRxiv a license to display the preprint in perpetuity. It is made available under aCC-BY 4.0 International license.

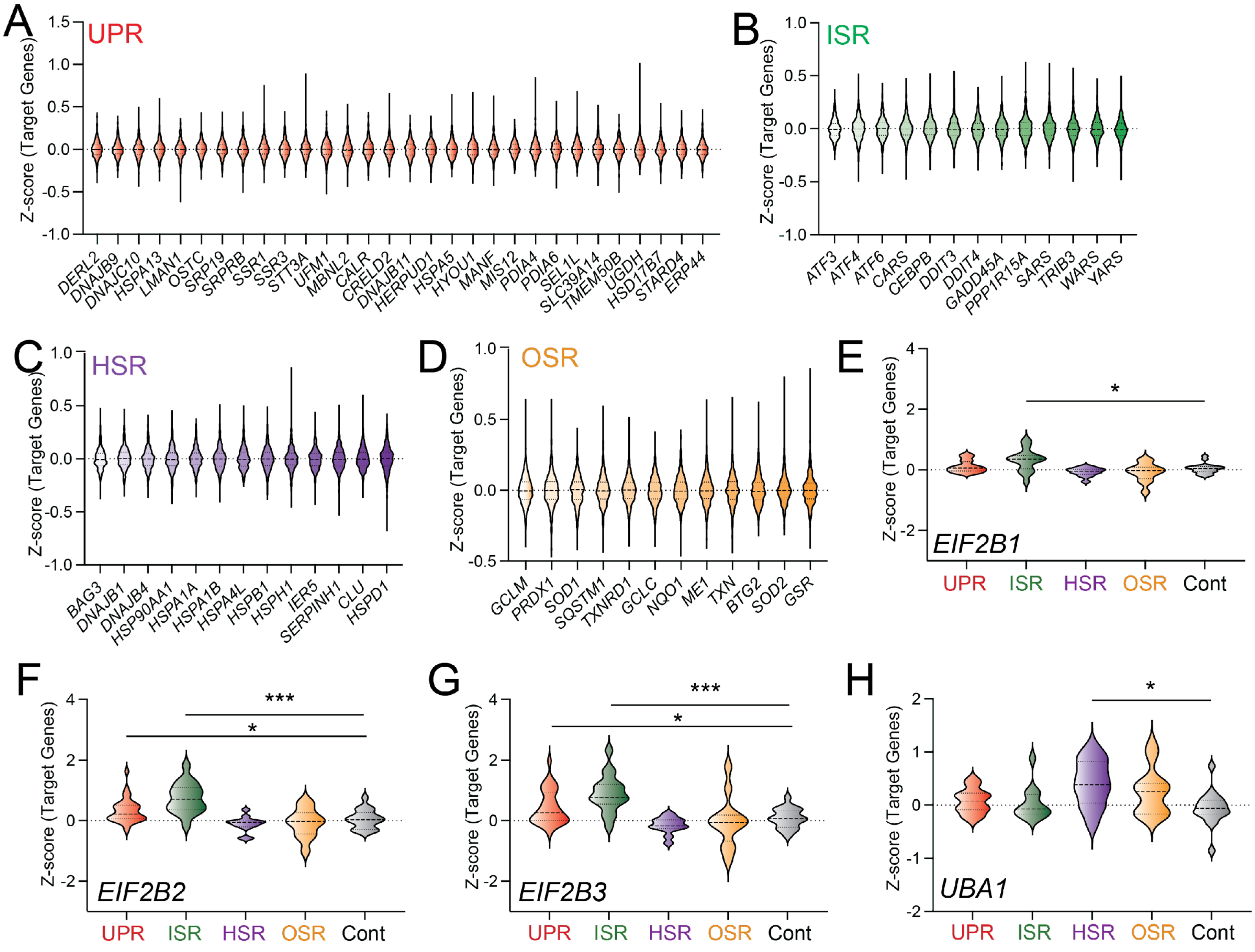

Figure S1 (Supplement to Figure 1). A-D. Expression, measured by z-score, of UPR (**A**), ISR (**B**), HSR (**C**), and OSR (**D**) in K562 cells expressing non-silencing guide RNAs. **E-H.** Expression, measured by z-score, of UPR, ISR, HSR, OSR, and control gene sets in K562 cells CRISPRi-depleted of *EIF2B1* (**E**), *EIF2B2* (**F**), *EIF2B3* (**G**), or *UBA1* (**H**). *p<0.05, ***p<0.005 for Brown-Forsythe and Welch ANOVA.

bioRxiv preprint doi: https://doi.org/10.1101/2024.01.30.577830; this version posted February 1, 2024. The copyright holder for this preprint (which was not certified by peer review) is the author/funder, who has granted bioRxiv a license to display the preprint in perpetuity. It is made available under aCC-BY 4.0 International license.

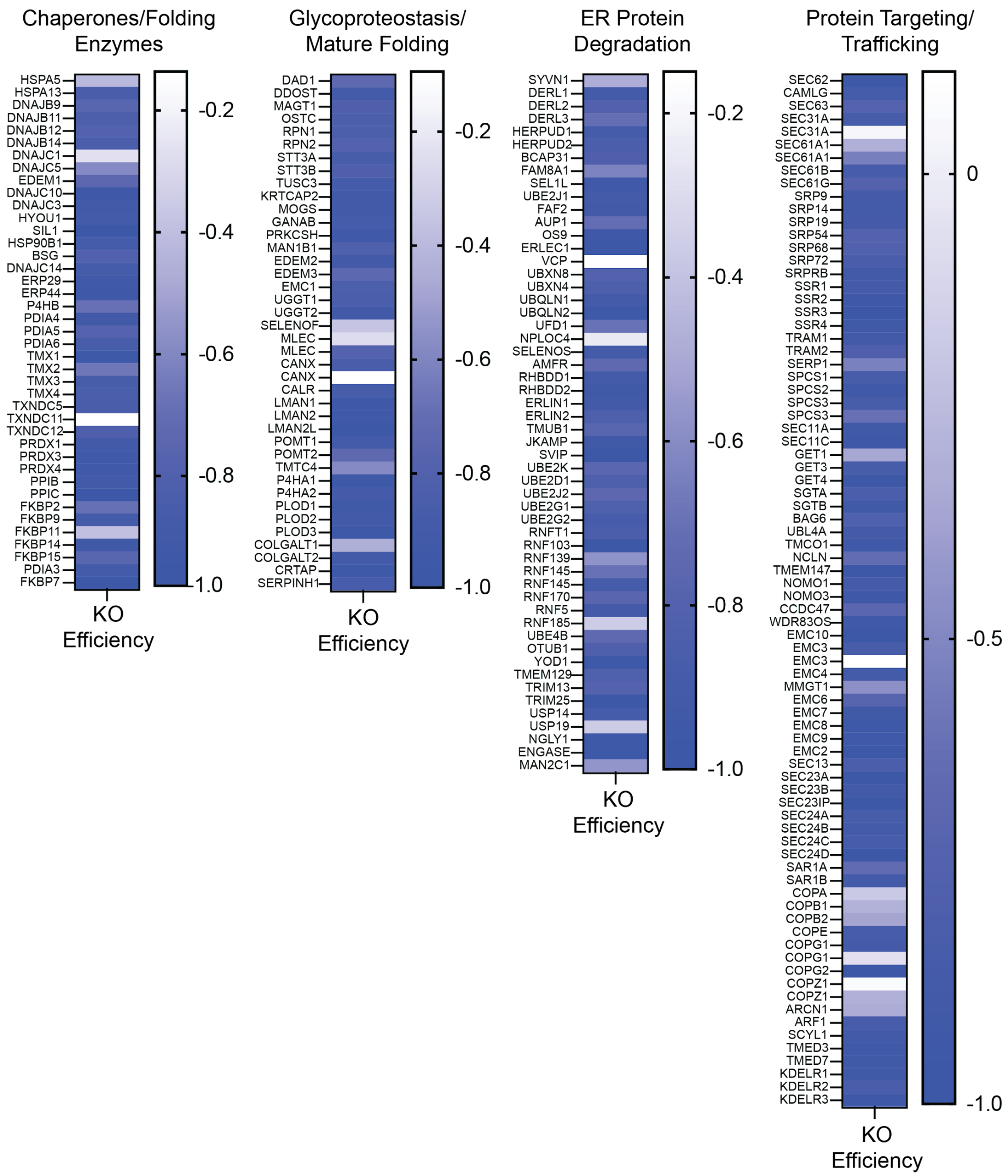

Figure S2 (Supplement to Figure 2). Knockdown efficiency of ER proteostasis genes. ER proteostasis genes were defined as in [1]. Knockdown efficiency was defined as in [42].

bioRxiv preprint doi: https://doi.org/10.1101/2024.01.30.577830; this version posted February 1, 2024. The copyright holder for this preprint (which was not certified by peer review) is the author/funder, who has granted bioRxiv a license to display the preprint in perpetuity. It is made available under aCC-BY 4.0 International license.

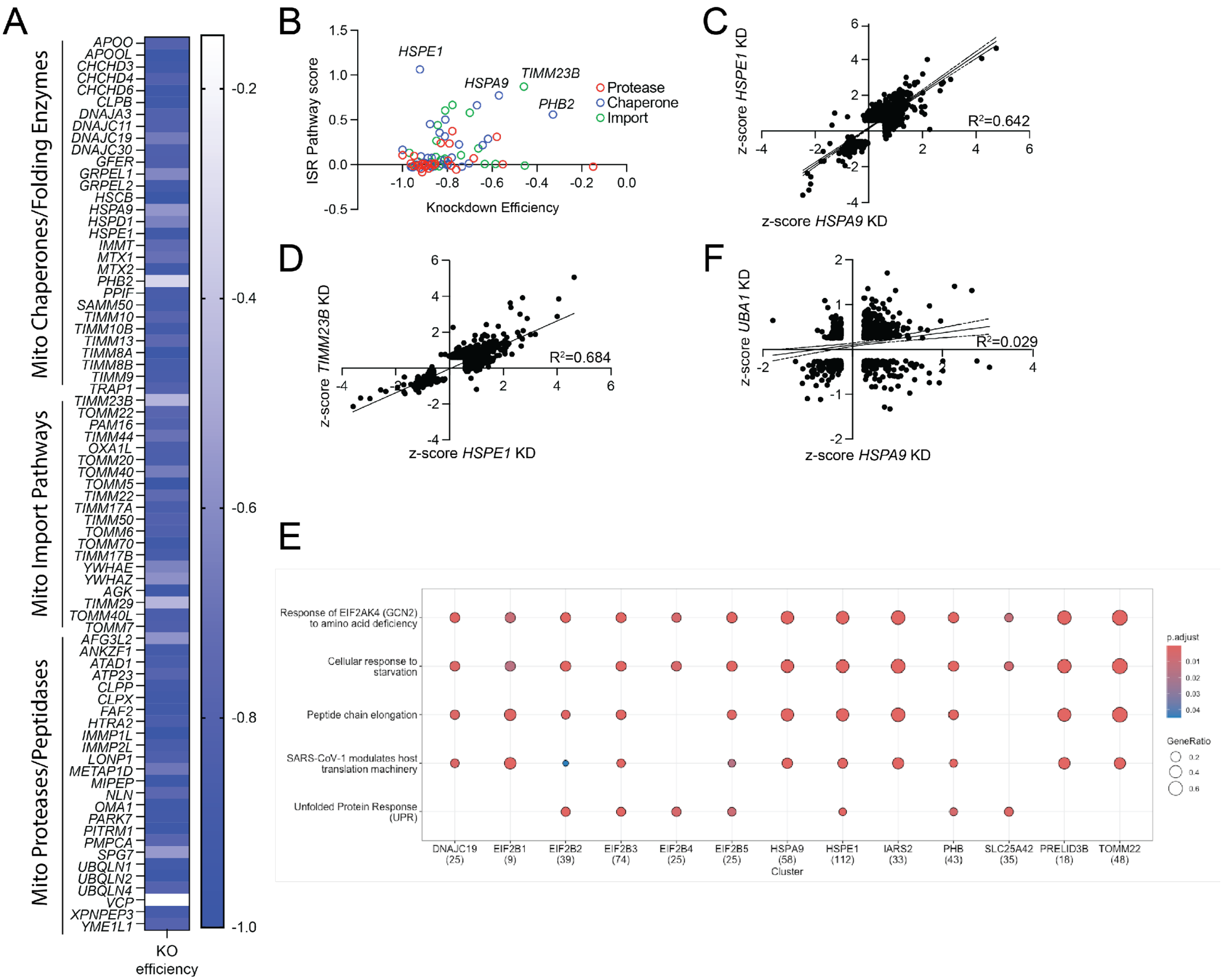

Figure S3 (Supplement to Figure 3). A. Knockdown efficiency of ER proteostasis genes. ER proteostasis genes were defined as in [1]. Knockdown efficiency was defined as in [42]. **B.** Plot of knockdown efficiency vs. ISR pathway score for mitochondrial proteostasis factors. IS activation score was defined by the average z-score for ISR target genes. **C,D.** Comparison of gene expression, as measured by z-score, in K562 cells CRISPRi-depleted of *HSPA9* vs *HSPE1* (**C**) or *TIMM23B* (**D**). Only genes increased or decreased |value| > 0.5 in response to either perturbation are shown. **E.** Comparison of ISR-related GO terms induced in K562 cells CRISPRi-depleted of the indicated mitochondrial gene. **F.** Comparison of gene expression, as measured by z-score, in K562 cells CRISPRi-depleted of *HSPA9* vs *UBA1*. Only genes increased or decreased |value| > 0.5 in response to either perturbation are shown.

bioRxiv preprint doi: https://doi.org/10.1101/2024.01.30.577830; this version posted February 1, 2024. The copyright holder for this preprint (which was not certified by peer review) is the author/funder, who has granted bioRxiv a license to display the preprint in perpetuity. It is made available under aCC-BY 4.0 International license.

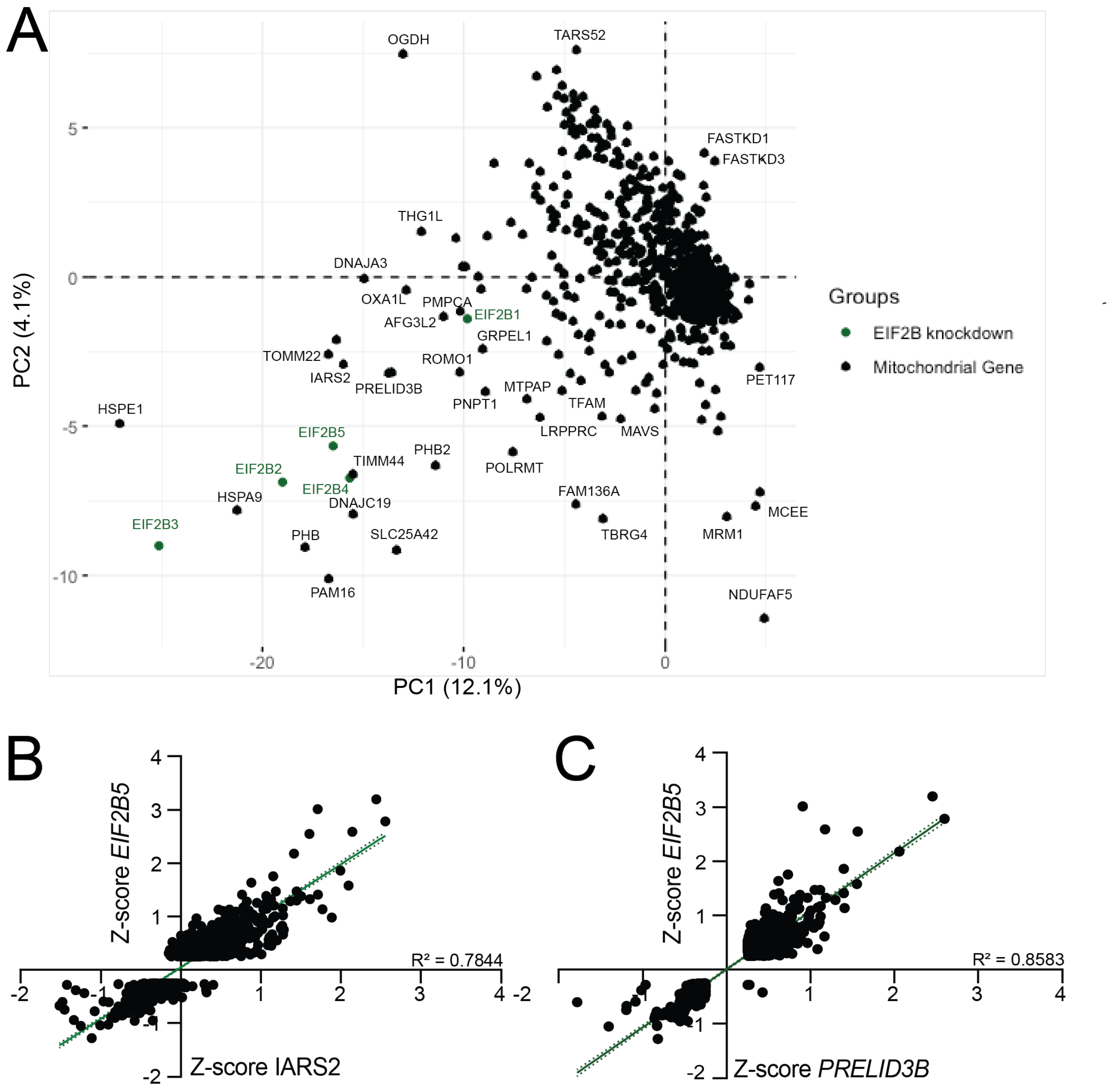

Figure S4 (Supplement to Figure 4). A. Principal component analysis (PCA) for gene expression observed in K562 cells CRISPRi-depleted of individual genes encoding mitochondrial proteins. Components of the EIF2B guanine exchange factor (*EIF2B1-5*) are also shown in green. **B**,**C.** Comparison of gene expression, as measured by z-score, in K562 cells CRISPRi-depleted of *IARS2* (**B**) or *PRELID3B* (**C**) vs *EIF2B*. Only genes increased or decreased |value| > 0.5 in response to either perturbation are shown.
